# Raman difference spectroscopy and U-Net convolutional neural network for molecular analysis of cutaneous neurofibroma

**DOI:** 10.1371/journal.pone.0302017

**Published:** 2024-04-11

**Authors:** Levi Matthies, Hendrik Amir-Kabirian, Medhanie T. Gebrekidan, Andreas S. Braeuer, Ulrike S. Speth, Ralf Smeets, Christian Hagel, Martin Gosau, Christian Knipfer, Reinhard E. Friedrich

**Affiliations:** 1 Department of Oral and Maxillofacial Surgery, University Medical Center Hamburg-Eppendorf, Hamburg, Germany; 2 Mildred Scheel Cancer Career Center HaTriCS4, University Medical Center Hamburg-Eppendorf, Hamburg, Germany; 3 Institute of Thermal-, Environmental- and Resources‘ Process Engineering, Technische Universität Bergakademie Freiberg, Freiberg, Germany; 4 Division of “Regenerative Orofacial Medicine”, Department of Oral and Maxillofacial Surgery, University Medical Center Hamburg-Eppendorf, Hamburg, Germany; 5 Institute of Neuropathology, University Medical Center Hamburg-Eppendorf, Hamburg, Germany; Indian Institute of Space Science and Technology, INDIA

## Abstract

In Neurofibromatosis type 1 (NF1), peripheral nerve sheaths tumors are common, with cutaneous neurofibromas resulting in significant aesthetic, painful and functional problems requiring surgical removal. To date, determination of adequate surgical resection margins–complete tumor removal while attempting to preserve viable tissue–remains largely subjective. Thus, residual tumor extension beyond surgical margins or recurrence of the disease may frequently be observed. Here, we introduce Shifted-Excitation Raman Spectroscopy in combination with deep neural networks for the future perspective of objective, real-time diagnosis, and guided surgical ablation. The obtained results are validated through established histological methods. In this study, we evaluated the discrimination between cutaneous neurofibroma (n = 9) and adjacent physiological tissues (n = 25) in 34 surgical pathological specimens *ex vivo* at a total of 82 distinct measurement loci. Based on a convolutional neural network (U-Net), the mean raw Raman spectra (n = 8,200) were processed and refined, and afterwards the spectral peaks were assigned to their respective molecular origin. Principal component and linear discriminant analysis was used to discriminate cutaneous neurofibromas from physiological tissues with a sensitivity of 100%, specificity of 97.3%, and overall classification accuracy of 97.6%. The results enable the presented optical, non-invasive technique in combination with artificial intelligence as a promising candidate to ameliorate both, diagnosis and treatment of patients affected by cutaneous neurofibroma and NF1.

## Introduction

Neurofibromatosis type 1 and 2 (NF1, NF2) and schwannomatosis make up the global spectrum of neurofibromatosis. With an incidence of one in 3,000, one in 33,000 and one in 60,000 births, respectively, they belong to the group of tumor suppressor gene diseases [1]. Herein, *NF1* is of autosomal dominant inheritance and the disease is characterized by neural, skeletal, and cutaneous abnormalities with a highly variable phenotype causing problems in several organ systems [2, 3]. Tumors of the nervous system are common in patients with NF1 [4]. In particular, numerous peripheral nerve sheaths tumors (PNST) are the hallmark of the disease [5]. PNST are caused by tumorous Schwann cells or Schwann cell precursors [6, 7]. The typical PNST in NF1 arises in the skin and is termed cutaneous or dermal neurofibroma. Neurofibromas are benign, heterogeneous peripheral nerve sheath tumors, arising from the connective tissue, occurring mostly as isolated sporadic lesions but occasionally as a consequence of genetic hereditary diseases [8]. Cutaneous neurofibromas typically only appear after puberty, usually develop in large numbers, reach a maximum size of a few centimeters, and are limited in growth to the skin and subcutaneous fat tissue [5]. On the other hand, PNST can develop in other regions, where they affect the main branches of larger nerves or grow invasively through entire regions of the body. This type of tumor is perceived to have developed in the embryonic or early postnatal phase. This is termed plexiform neurofibroma (PNF). Due to clinical and morphological differences, descriptive terms such as "nodular" or "diffuse" and combinations of these adjectives are used to describe the heterogenous morphology of PNF [5]. Both cutaneous and PNF do not differ in the mutation spectrum of the *NF1* gene [9]. In contrast, tumor biology of NF1-associated PNST is clearly differentiated. While cutaneous neurofibromas may represent significant aesthetic, painful or functional problems in individual locations, they do not impose a tendency for malignant transformation [10]. PNF on the other hand is a facultative precancerosis that may give rise to a malignant peripheral nerve sheath tumor (MPNST) [11, 12]. The life-time risk of an NF1 patient to develop MPNST amounts to approximately 10% which is associated with a poor prognosis [13, 14].

Adequate diagnosis and treatment require close interdisciplinary cooperation between dermatologists, neurologists, surgeons, radiologists, pathologists, other medical specialists, as well as pediatricians or general practitioners. In conditions where patients are compromised, treatment often requires surgical removal of neurofibroma. Herein, visual identification of adequate surgical resection margins is a pivotal step in therapy which harbors difficulties. A major challenge is the absence of a capsule or the surrogate of a pseudo-capsule. Consequently, after histopathological examination, residual tumor extension beyond marginal sections may frequently be observed. As some tumors of NF1 patients cannot be removed completely, there is considerable interest in molecular-targeted drug therapy capable to slow down growth of tumors or even tumor shrinkage [15]. The recent regulatory approvals (including United States and Europe) of the mitogen-activated protein kinase kinase (MAPKK or MEK) inhibitor selumetinib for children with NF1 and symptomatic, inoperable plexiform neurofibromas (PN), have opened new therapeutic approaches in the management of PNs [16]. Detailed knowledge of cellular metabolism, however, is essential for rational chemotherapy of neurofibromas and MPNST [17]. The prerequisite for the application of this therapeutic concept is the determination of the morphological composite in the tumors and, in the case of heterogeneous tumor populations, the identification of cellular differences as well as to establish non-invasive monitoring of the targeted pathologic lesions.

In a translational approach, optical techniques have the potential to enable objective, non-contact tissue identification in real-time. Raman spectroscopy is a molecular spectroscopic technique capable of providing detailed information about the chemical and molecular composition of biological tissue [18]. Despite best efforts in treatment of NF1, the clinical course of the disease is often characterized by recurrence, as unrecognized tumor tissue may lead to progression of the lesion. As resection remains subjective and relies largely on individual experience, there is an unmet demand for an objective method for the precise identification of neurofibromas and guided surgical ablation. Herein, we introduce Shifted-Excitation Raman Difference Spectroscopy (SERDS) with U-Net deep neural network for spectrum purification as a promising candidate [19]. With improving non-invasive diagnosis and adequate surgical resection margin indication in the future, this technique has the potential to ameliorate the overall clinical outcome of patients affected by this disease.

## Materials and methods

### Study collective and sample acquisition

**Fig 1:**
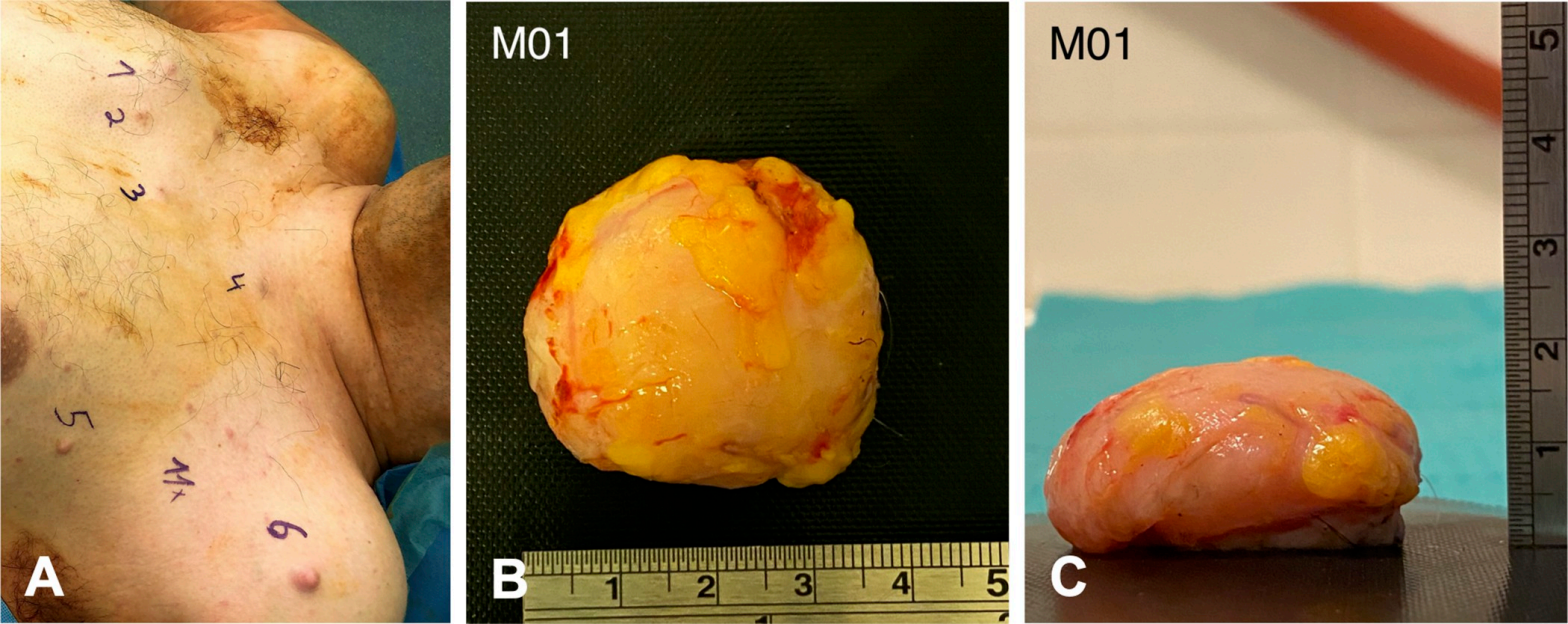
Clinical presentation and macroscopic aspect of cutaneous neurofibroma. (A) Example of a 43-year-old male patient that underwent surgical removal of eleven neurofibromas on the trunk and extremities (not shown), nine of which were processed for spectral analysis. (B, C) Macroscopic aspect of representative surgical specimen measuring 3.7x1.8 cm from top and lateral view.

A total of 20 patients were enrolled at the Department of Oral and Maxillofacial Surgery at the University Medical Center Hamburg-Eppendorf in 2020. Patients underwent surgical tissue removal as indicated by tumor burden (see Fig 1). All surgical resections were performed by the same experienced surgeon. After anatomical orientation, Raman spectra were collected from the specimens *ex vivo*, which were subsequently confirmed histologically. Physiological tissue, obtained from the biobank archive of the department, served as controls: skin, mucosa, bone, nerve, and fat. Herein, data collection was performed after pseudonymization, without individual participant identification. All tissue samples were kept in their physiologic state without fixation medium (isotonic 0.9% NaCl solution to prevent drying) and processed for spectroscopic analysis within a maximum time of four hours. For each specimen, in average three evenly distributed measurement locations were chosen. Following histopathological confirmation, the spectroscopic data were referenced to the corresponding tissue entities. The sample collection and experimental study was conducted from 2020 through 2021. Patients’ written informed consent was obtained before participation. The study is in accordance with the 1964 Declaration of Helsinki and its later amendments and has been approved by the research ethics committee of the University of Hamburg (AZ PV7012, 11.02.2020).

### Technical set-up

**Fig 2:**
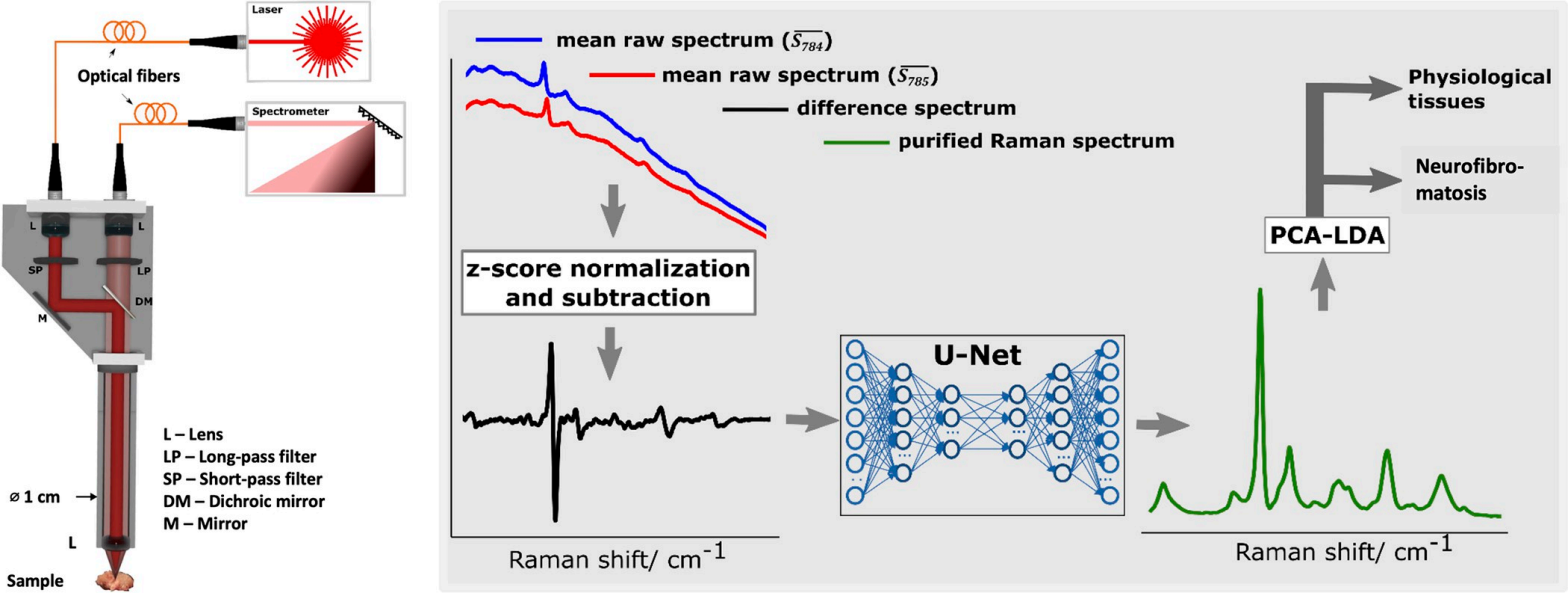
Illustration of Raman spectral acquisition and processing. Left panel: Sketch of self-engineered Raman probe as described in [20]. DM: dichroic mirror; L: lens; LP: long-pass filter; M: mirror; SP: short-pass filter; Right panel: Illustration of efficient extraction of Raman spectrum from extreme fluorescence interference and biological tissues classification.

A self-engineered Raman probe connected to a spectrometer and a laser was used as previously described in detail [20, 21]. In brief, an excitation laser beam (power 115 mW) is guided from the diode laser (Littman/Metcalf, Sacher Lasertechnik, Marburg, Germany) through an optical fiber (200 μm core diameter, 0.22 NA) to the Raman probe. After passing of a short pass filter (780/12 Brightline HC, Semrock, Rochester, New York, USA), a dichroic mirror and an achromatic lens (THORLABS, Newton, New Jersey, USA), the laser beam is focused onto the specimen. The inelastically scattered Raman signal is collected, together with undesired background interferences (elastically scattered signals and laser-induced fluorescence). A dichroic mirror and a long-pass filter suppress the elastically scattered signal. As described, fluorescence and Raman signals pass the dichroic mirror towards another lens focusing them onto a detection glass fiber (600 μm core diameter, 0.37 NA) guiding the signals from the Raman probe to the spectrometer (Ventana-785-Raman, Ocean Optics, Rochester, New York, USA) (see Fig 2). At 1000 ms acquisition time, the spectrometer acquires the spectra from 800 to 940 nm, equivalent to Raman shifts from 200 to 2000 cm^-1^. The focal spot position was optimized for maximum signal intensity through z-axis translation in every measurement locus, resulting in a working distance of approximately 10–11 mm. The acquired Raman spectra are expected to be collected from a tissue surface layer of approximately 100 μm in depth, thus the Raman signal is derived primarily from the epithelial aspects [22].

### Data processing

Routinely, for each tissue measurement locus, 50 spectra *S*_784_ were acquired using the excitation wavelength of 784 nm. Then, another 50 spectra *S*_785_ were recorded at 785 nm. Mean spectra $\bar{S_{784}}$ and $\bar{S_{785}}$ were averaged from each of the 50 raw spectra. Extraction of the pure Raman spectra from the mean raw spectra, which were heavily interfered by autofluorescence, was performed using the SERDS and U-Net deep neural network data processing methods developed in-house by our workgroup for biological tissue specimens [19–21]. Here, it is briefly explained in the context of Fig 2. The mean raw spectra $\bar{S_{784}}$ and $\bar{S_{785}}$ in blue and red are first z-score normalized, then a difference spectrum is obtained (black curve). This difference spectrum is provided as an input to the U-Net convolutional neural network to obtain a purified Raman spectrum as an output (green curve) [19]. This method provides better (completely background free) purified Raman spectra than the standard (without U-Net) SERDS Raman spectrum recovery approach.

In order to differentiate between neurofibroma and physiological tissues based on their Raman spectra (Raman shifts between 550 to 1800 cm^-1^), we employed a multiclass linear discriminant analysis (LDA) [23]. As a first step, principal component analysis (PCA) was utilized to reduce the number of spectral parameters by generating a new set of independent features ordered by the largest variability in the dataset [24]. As previously described in detail, in every iteration a PCA was performed separately for each cross-validation [21]. Discrimination between the different measurement loci, specimens and types of tissue entities was made by splitting the data set into training (80%) and test (20%) data sets according to a 5-fold cross validation [25]. Once trained, the deep learning approach may be applied for real-time SERDS data analysis.

### Histology

Following spectroscopic analysis, tissue samples were processed and validated histologically in the Institute of Neuropathology at the University Medical Center Hamburg-Eppendorf. Regions of spectral data acquisition were routinely fixed in 3.7% PBS-buffered formaldehyde, dehydrated with ethanol, embedded in paraffin, and sections performed on a microtome. Four standard 10-μm-thick sections were stained with Hematoxylin & Eosin for each specimen. For this study, the material was evaluated by the same experienced neuropathologist applying current WHO criteria to diagnose peripheral nerve sheath tumors [26].

## Results

For this study, raw Raman spectra were acquired from 82 distinct measurement loci in 34 surgical specimens of cutaneous neurofibroma and physiological tissues of nerve, fat, skin, bone, and mucosa. Raw Raman spectra of neurofibroma were collected from nine samples in one patient to circumvent inter-individual variability. For the physiological tissues, a total of 73 measurement loci from 19 patients were included. Tissue entity, respective number of specimens and spectroscopic measurement loci were as follows: cutaneous neurofibroma (9, 9), physiological skin (4, 12), nerve (2, 6), fat (5, 13), bone (5, 15), and mucosa (9, 27).

**Fig 3:**
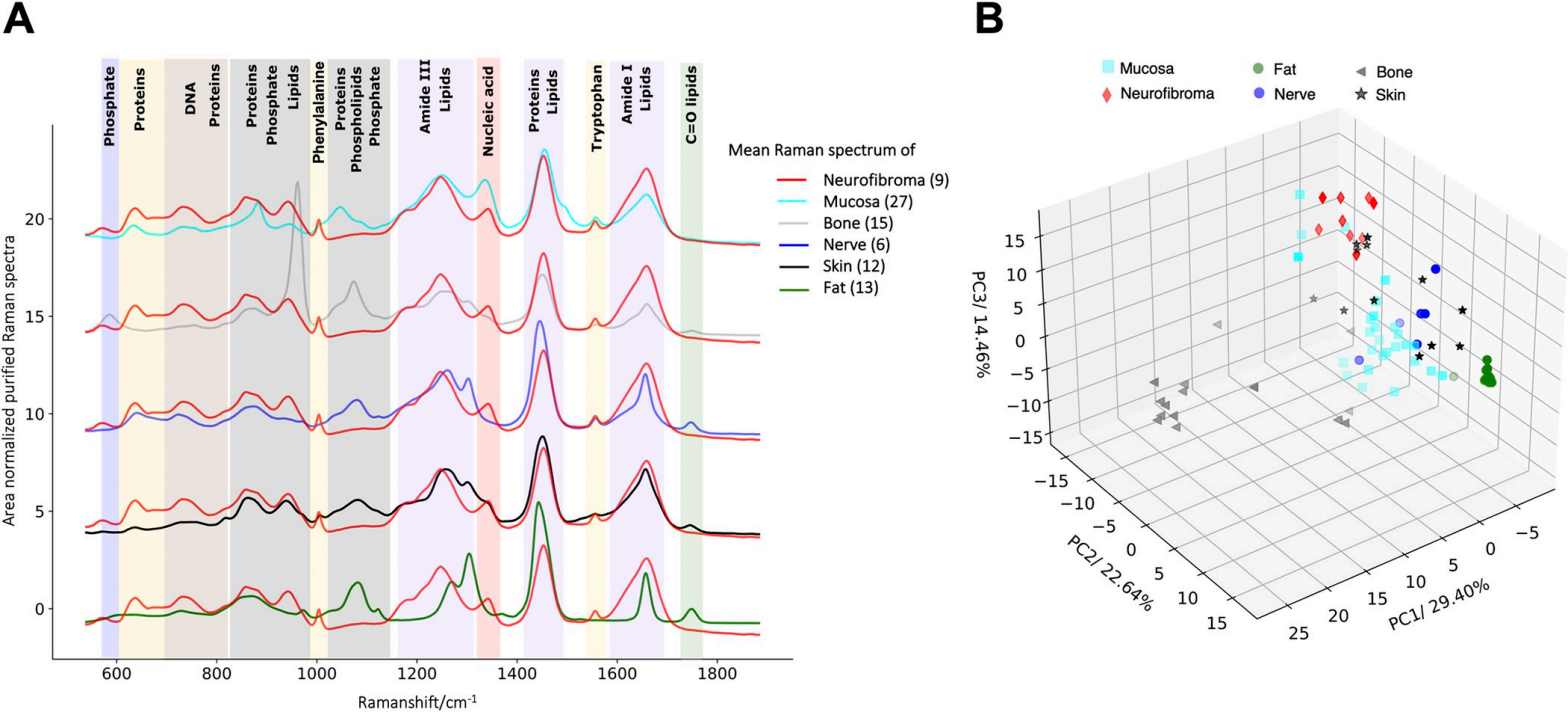
Distinction of neurofibroma from other physiological entities via Raman spectroscopy and PCA. (A) Mean purified spectra of cutaneous neurofibroma, physiological skin, fat, nerve, bone and mucosa, and the assignment of the Raman peaks to their respective molecular origin is shown in rectangular colorized boxes. (B) PCA with scatter plot of the projections of the purified Raman spectra of cutaneous neurofibroma, as well as physiological skin, fat, nerve, bone, and mucosa onto the first three principal components.

After application of U-Net for the refinement from background interferences, the mean purified Raman spectra of cutaneous neurofibroma and physiological mucosa, bone, nerve, skin, and fat are shown (see Fig 3A). For reference and comparison, the mean Raman spectra of each physiological tissue are plotted against the mean Raman spectrum of the cutaneous neurofibroma. Molecular origin of the spectral Raman signatures is displayed as annotations. The majority of the mean Raman spectra peaks of fat and nerve tissue feature signatures of lipid molecules. However, the mean Raman spectrum of cutaneous neurofibroma reflects distinct protein signatures such as tryptophan, phenylalanine as well as peaks derived from broader Amide I and Amide III. It also shows a clear nucleic acid signature. The mean purified Raman spectrum of bone reflects prominent molecular signatures of phosphate around 960 cm^-1^, absent in other tissue entities. The Raman spectra of the neurofibromas reflect a significant contribution from protein molecules. As determined by loading plots and available literature, the assigned molecular origins are depicted in Table 1.

**Table 1:** Assignment of spectral features in the mean Raman spectrum to the molecular vibrations [18, 27–32].

Raman Shift [cm^-1^]	Molecular assignment
937/8	Proline, Hydroxyproline, C-C stretching of collagen backbone
1004	Phenylalanine (of collagen)
1038	Phenylalanine (of collagen)
1073	Proline (collagen assignment), Glucose, Triglycerides, C-C (lipid)
1095	DNA, C-N stretching
1126	C-N stretching
1160	C-C Stretching of the carotenoid polyene chain
1200–1320	Amide III region
1338, 1340	CH_2_ deformation (Protein, A and G of DNA/RNA), nucleic acid
1378	CH_3_ in-phase deformation (T, A, G of DNA)
1440–1470	C─H deformation arising from CH_2_ and CH_3_ of lipid and protein
1532	Amide II, carotenoid peak
1600–1670	Amide I region

In order to determine the differentiability of the neurofibroma tissue from physiological tissues, we applied PCA. Fig 3B shows the scatter plot of the projections of the Raman spectra on the first three principal components. Herein, the first principal component (PC) accounts for 29.4%, PC 2 comprises 22.6% and principal component three amounts to 14.5% of spectral data variation. As visualized, distinct differences between the purified Raman spectra of neurofibromas and the purified Raman spectra of fat, nerve, and bone tissues occurred. However, some of the Raman spectra of skin and mucosa appear to be closer to the spectra of neurofibroma and overlap to a minimal extent.

For the differentiation of cutaneous neurofibroma from the physiological tissues, a PCA-LDA classifier was applied. Table 2 summarizes the classification results. These reveal that neurofibroma can be differentiated from physiological tissues with a sensitivity of 100%, specificity of 97.3% and an overall accuracy of 97.6% with the proposed method. Two Raman spectra of physiological tissue were misclassified as Raman spectra of neurofibroma tissue, resulting in a 2.4% classification error. The receiver operating area under the curve (ROC-AUC) was calculated as 97.9%. The purified Raman spectra of neurofibroma were perfectly classified against the Raman spectra of physiological fat, bone, and nerve tissues (sensitivity, specificity, and overall accuracy of 100%). The Raman spectra of mucosa were classified with a specificity of 92.6% and sensitivity of 100% against the Raman spectra of neurofibroma, with an overall accuracy of 94.4% and AUC of 98.0%. Of 27 specimens, two mucosal tissue samples were misclassified as cutaneous neurofibroma, whereas nine of nine NF samples were correctly classified. The classification between the purified Raman spectra of skin and neurofibroma were calculated with a sensitivity of 88.9% and specificity of 100%. One Raman spectrum of neurofibroma was misclassified as skin tissue. The data analysis gave the respective classification error of 4.8% with an overall accuracy of 95.2%.

**Table 2:** Classification results of cutaneous neurofibroma against all physiological tissue. The performance of the classification estimated based on sensitivity, specificity, overall accuracy and soon.

Neurofibroma vs	All	Fat	Bone	Nerve	Mucosa	Skin
**Sensitivity**	100%	100%	100%	100%	100%	88.9%
**Specificity**	97.3%	100%	100%	100%	92.6%	100%
**Accuracy**	97.6%	100%	100%	100%	94.4%	95.2%
**Classification Error**	2.4%	0%	0%	0%	5.6%	4.8%
**AUC**	97.9%	100%	100%	100%	98.0%	95.0%
**Confusion Matrix** TP FP FN TN	$\begin{array}{cc} 71 & 2\\ 0 & 9 \end{array}$	$\begin{array}{cc} 13 & 0\\ 0 & 9 \end{array}$	$\begin{array}{cc} 15 & 0\\ 0 & 9 \end{array}$	$\begin{array}{cc} 6 & 0\\ 0 & 9 \end{array}$	$\begin{array}{cc} 25 & 2\\ 0 & 9 \end{array}$	$\begin{array}{cc} 12 & 0\\ 1 & 8 \end{array}$

AUC area under the curve, TP true positive, TN true negative, FP false positive, FN false negative

**Fig 4:**
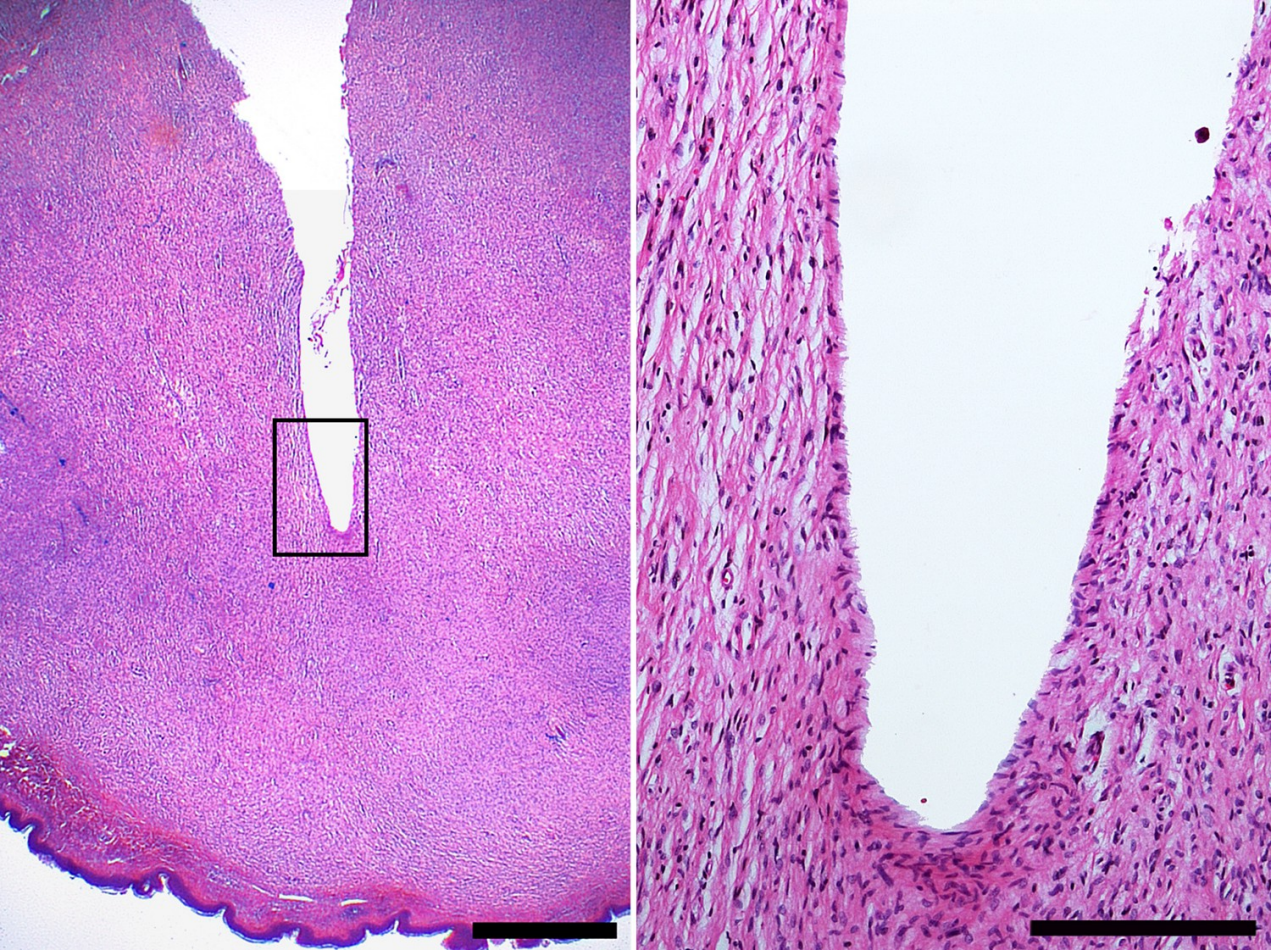
Histology of cutaneous neurofibroma in the plane examined by Raman spectroscopy. Left, cutaneous neurofibroma presenting as tumor with uniform cell density extending to the subepidermal tissue (bottom of the image). Note the defect set by the needle which was inserted to mark the plane examined by Raman spectroscopy. Hematoxylin & Eosin stain, scale bar 1 mm. Right, close-up of the framed area in the left picture depicting spindle shaped tumor cells with wavy contour and slender nuclei. Hematoxylin & Eosin stain, scale bar 200 μm.

In order to confirm and validate this technique, specimens were marked with a needle after spectra acquisition and further examined by histology. To this end, sections were stained with Hematoxylin & Eosin. Here, cutaneous neurofibroma was confirmed by uniform density of spindle shaped tumor cells with wavy contour and slender nuclei. No mitosis or necrosis was seen. Representative images are shown in Fig 4.

## Discussion

Advances in molecular biology have provided new insights into the nature of the various peripheral nerve sheath tumors [33]. Recently, preoperative differentiation of benign and malignant peripheral nerve sheath tumors in neurofibromatosis type 1 based on MRI has been shown with good results (sensitivity 94%, specificity 97%) [34]. But as of now, intraoperative discrimination of benign neurofibroma from physiological tissue, or even malignancy, remains largely subjective. To improve this, non-invasive characterization of molecular tissue composition and diagnosis in real time is desirable and here, Raman spectroscopy bears great potential [35–37].

For the safe and effective removal of central nervous system tumors, navigation, fluorescence-guided surgery, and intraoperative structural imaging, including CT and MRI, have led to improved accuracy in resection volumes and patient outcomes [38, 39]. While most of these methods are indispensable in neurosurgical oncology, a limitation remains the need for contrast agents such as gadolinium or fluorescent dyes like 5-aminolevulinic acid [40]. Fluorescence-guided surgery and MRI-based methods cannot resolve tumor infiltration at the microscopic level, whereas vibrational spectroscopy, which includes infrared and Raman spectroscopy, can detect tumor infiltration directly based on the molecular properties of the tissue and can be used without additional labeling agents [38]. Raman spectroscopy has been proven that it has potential in the evaluation of tumors of the central nervous system [30, 41]. But the technique has also been shown to be useful for the characterization of peripheral nerve imaging. With the shared goal of potential intraoperative application and nerve-sparing surgery, various optical techniques have been applied for the visualization of peripheral nerves and discrimination against adjacent tissues. For example, fluorescent peptides specifically labeling peripheral nerves [42], spontaneous Raman scattering [43], coherent anti-Stokes Raman scattering (CARS) [44] or combination of autofluorescence and second harmonic generation [45, 46]. In conjunction with immunohistochemistry and Raman spectroscopy, Morisaki et al. were able to evaluate changes in axon and myelin turnover for the analysis of peripheral nerve regeneration after sciatic nerve injury in rats [47]. Kumamoto et al. differentiated peripheral nerve bundles from adjoining connective tissue and muscle with a sensitivity, specificity and accuracy of 85.8%, 96.0%, and 90.8%, respectively, in an *ex vivo* rat model [48]. But to date, there is limited evidence concerning the evaluation of peripheral nerve sheath tumors in humans. Rabah et al. have successfully applied Raman spectroscopy to diagnose neuroblastoma and ganglioneuroma in children [49]. Here, the spectral profile of neuroblastoma was compared to nerve sheath tumors with the finding, that the nerve sheath tumors have a similar collagenous schwannoma stromal configuration compared to maturing neuroblastomas and ganglioneuromas. They also described that nerve sheath tumors exhibited increased collagen and protein contents which are attributable to spectral features appearing at 854, 938, 1003, 1447, and 1657 cm^-1^ and a shoulder in the Amide III region (1240–1270 cm^-1^). Consecutively, the same group of authors applied Raman spectroscopy to detect and distinguish neuroblastoma, nerve sheath tumor and related tissues in fresh and frozen specimens *ex vivo* [50]. They reported the ability of Raman spectroscopy to accurately identify cryopreserved tissue specimens.

Application of Raman spectroscopy has been shown successful in the analysis of skin composition, skin penetration studies for the evaluation of pharmaceutical formulation and skin diseases [51]. Among these are atopic dermatitis, psoriasis, benign neoplasms, and malignancies such as basal cell carcinoma, squamous cell carcinoma or melanoma [52]. Our results show for the first time, that cutaneous neurofibroma can be distinguished from a variety of other human physiological tissues with an overall accuracy of 97.6% in our collective based on reproducible Raman spectral peaks which can be assigned to its molecular origin. Combination of Raman spectroscopy with machine learning algorithms has been shown in a variety of medical and non-medical applications [51]. To the best of our knowledge, our group is the first to employ SERDS in conjunction with deep neural networks for spectral processing and PCA-LDA for data classification in this field of research. The SERDS technique has been shown useful in reducing fluorescence interference, inherent to biologic tissues [53–57]. The method has been described previously by our group in detail but can be explained in brief as follows [20, 21, 56, 58]. This technique was first proposed by Shreve and colleagues and has been established as a useful tool for the application of Raman spectroscopy to samples with strong fluorescence interferences, such as viable biological tissue [53–56]. Kasha discovered that the fluorescence signal is almost insensitive to small changes in excitation photon energy, unlike the Raman spectrum, which shifts depending on the difference in excitation photon energy [59]. By subtracting two raw spectra, each excited with a slightly different photon energy, the fluorescence background can be eliminated, leaving a Raman difference spectrum. SERDS efficiently eliminates both fluorescence interference and systematic background, such as etaloning, from spectra without any sample preparation [60, 61]. A variety of approaches for the reduction of autofluorescence exists, which can be divided into mathematical [62, 63] and physical methods [64]. The principal advantage of SERDS is that fluorescence is eliminated primarily because of a physical approach, which in contrast to mathematical based protocols, does not alter the Raman spectral features [20, 65]. Introduction of charge-shifting spectral detection has been shown to result in a more even distribution of the fluorescence and hence a lower residual background in the difference spectra [66, 67]. Our group was the first to apply SERDS followed by U-Net deep neural network data processing for the refinement of Raman spectra from background interferences in biological tissue, especially in pathologies of the head & neck region [19]. The U-Net architecture originally described by Ronneberger and colleagues for medical image segmentation was adopted for one-dimensional Raman spectral analysis in this study [68]. As raw Raman spectra contain considerable amounts of noise and background, these need to be refined to obtain the desired signal. In brief, the network architecture consists of an encoder network followed by a decoder. It uses a network of convolutional layers to predict the desired output from noise and background disturbed raw spectra (input). The combination of SERDS with deep neural network processing reduces both, fluorescence interference and systematic background noise, without the need of labeling, fixation, staining or any additional sample preparation *ex vivo* [60, 61]. Of emphasis is that specimens were analyzed in their physiological state (isotonic 0.9% NaCl solution), without the addition of labeling agents preoperatively which make the results of the study readily available for the transfer from bench to bedside in the near future.

As the current gold-standard and constituting reference, each neurofibroma specimen was examined by histology. A disadvantage is the significantly longer processing time and associated time to diagnosis, which precludes its direct intraoperative use and therefore makes our translational approach particularly interesting. Recently, Raman histology has been shown to be a promising complement for *in vivo* skin cancer and brain tumor diagnosis [52, 69]. Here, to validate the optical method, analysis by means of histology with Hematoxylin & Eosin stainings was conducted. Despite tissue fixation via formaldehyde solution being beneficial for histological examination, it is known to influence spectroscopic properties [70, 71]. Lastly, the setup presented is feasibly applicable under ambient light, as the SERDS technique can effectively eliminate the interference of room light, making it a promising candidate for application in the operating theatre [72–74].

The *NF1* gene and neurofibromin regulate keratinocyte differentiation and melanin synthesis [3]. The *NF1* gene furthermore plays a pivotal role in tumorigenesis. Recent studies confirmed that Schwann cells initiate neurofibroma formation and such tumors associated with neurofibromatosis 1 show a loss of *NF1* gene expression and high levels of Ras [8]. These may be future targets for the detection and quantification via Raman spectroscopy. Another candidate are components of collagen [75]. The main contribution of the collagen component to the Raman protein signatures could be due to neurofibroma consisting of connective tissues and collagen to a significant proportion [8]. Rabah et al. have previously observed collagen spectral features in the Raman spectra of nerve sheath tumors [49]. However, the specificity of collagen to the peripheral nerve sheath tumors in contrast to physiological connective tissue constituents remains a subject of ongoing investigation.

Despite interesting findings, limitations with this study remain. Measurements were performed *ex vivo* and the specimens were derived from a limited number of individuals. Development of a mobile system with *in vivo* spectra acquisition intraoperatively is desirable and currently being pursued. In the further course of the project, insights are expected that allow the marginal area to be distinguished with the goal of adequate surgical resection indication to eliminate residual or prevent recurrent neurofibroma. Furthermore, we aim at investigating into features of neurofibroma sub-entities (e.g., diffuse, nodular, plexiform) in a larger study collective with the support of convolutional neural networks.

## Conclusions

Herein, Raman spectra of various tissues were isolated with the SERDS technique in conjunction with U-Net convolutional neural network. The distinction of cutaneous neurofibroma from physiological tissues was feasible with high sensitivity, specificity, and overall accuracy in this collective. By broadening the understanding of molecular changes, including mapping with deep learning data processing–for the first time in this field of research–we aim to improve the accurate evaluation of cutaneous neurofibroma and ultimately enhance both diagnosis and treatment of patients affected by this disease.

## References

[1] Farschtschi S, Mautner VF, McLean ACL, Schulz A, Friedrich RE, Rosahl SK. The Neurofibromatoses. Dtsch Arztebl Int. 2020;117(20):354–60. doi: 10.3238/arztebl.2020.0354 32657748 PMC7373809

[2] Legius E, Marchuk DA, Collins FS, Glover TW. Somatic deletion of the neurofibromatosis type 1 gene in a neurofibrosarcoma supports a tumour suppressor gene hypothesis. Nat Genet. 1993;3(2):122–6. doi: 10.1038/ng0293-122 8499945

[3] Peltonen S, Kallionpaa RA, Peltonen J. Neurofibromatosis type 1 (NF1) gene: Beyond cafe au lait spots and dermal neurofibromas. Exp Dermatol. 2017;26(7):645–8. 27622733 10.1111/exd.13212

[4] Gutmann DH, Ferner RE, Listernick RH, Korf BR, Wolters PL, Johnson KJ. Neurofibromatosis type 1. Nat Rev Dis Primers. 2017;3(1):17004. 28230061 10.1038/nrdp.2017.4

[5] Scheithauer BW, Woodruff JM, Erlandson RA. Tumors of the peripheral nervous system: Amer Registry of Pathology; 1999.

[6] Kluwe L, Friedrich R, Mautner VF. Loss of NF1 allele in Schwann cells but not in fibroblasts derived from an NF1-associated neurofibroma. Genes Chromosomes Cancer. 1999;24(3):283–5. doi: 10.1002/(sici)1098-2264(199903)24:3&lt;283::aid-gcc15&gt;3.0.co;2-k 10451710

[7] Kluwe L, Friedrich RE, Mautner VF. Allelic loss of the NF1 gene in NF1-associated plexiform neurofibromas. Cancer Genet Cytogenet. 1999;113(1):65–9. doi: 10.1016/s0165-4608(99)00006-0 10459349

[8] Ferner RE, O’Doherty MJ. Neurofibroma and schwannoma. Curr Opin Neurol. 2002;15(6):679–84. doi: 10.1097/01.wco.0000044763.39452.aa 12447105

[9] Upadhyaya M, Spurlock G, Monem B, Thomas N, Friedrich RE, Kluwe L, et al. Germline and somatic NF1 gene mutations in plexiform neurofibromas. Hum Mutat. 2008;29(8):E103–11. doi: 10.1002/humu.20793 18484666

[10] Friedrich RE, Nornberg LKN, Hagel C. Peripheral nerve sheath tumors in patients with Neurofibromatosis Type 1: morphological and immunohistochemical study. Anticancer Res. 2022;42(3):1247–61. doi: 10.21873/anticanres.15592 35220215

[11] McCarron KF, Goldblum JR. Plexiform neurofibroma with and without associated malignant peripheral nerve sheath tumor: a clinicopathologic and immunohistochemical analysis of 54 cases. Mod Pathol. 1998;11(7):612–7. 9688181

[12] Simsek FS, Akarsu S, Narin Y. Can we differentiate malignant peripheral nerve sheath tumor from benign neurofibroma without invasive sampling. World J Nucl Med. 2019;18(1):66–8. doi: 10.4103/wjnm.WJNM_11_18 30774551 PMC6357707

[13] Evans DG, Huson SM, Birch JM. Malignant peripheral nerve sheath tumours in inherited disease. Clin Sarcoma Res. 2012;2(1):17. doi: 10.1186/2045-3329-2-17 23036231 PMC3499223

[14] Combemale P, Valeyrie-Allanore L, Giammarile F, Pinson S, Guillot B, Goulart DM, et al. Utility of 18F-FDG PET with a Semi-Quantitative Index in the Detection of Sarcomatous Transformation in Patients with Neurofibromatosis Type 1. PLoS One. 2014;9(2):e85954. doi: 10.1371/journal.pone.0085954 24516522 PMC3916322

[15] Burki TK. Selumetinib for children with plexiform neurofibromas. Lancet Oncol. 2017;18(2):e69. doi: 10.1016/S1470-2045(17)30009-8 28089105

[16] Fisher MJ, Blakeley JO, Weiss BD, Dombi E, Ahlawat S, Akshintala S, et al. Management of neurofibromatosis type 1-associated plexiform neurofibromas. Neuro Oncol. 2022;24(11):1827–44. doi: 10.1093/neuonc/noac146 35657359 PMC9629437

[17] Reilly KM, Kim A, Blakely J, Ferner RE, Gutmann DH, Legius E, et al. Neurofibromatosis Type 1-associated MPNST state of the science: outlining a research agenda for the future. J Natl Cancer Inst. 2017;109(8). doi: 10.1093/jnci/djx124 29117388 PMC6057517

[18] Verdiyan EE, Allakhverdiev ES, Maksimov GV. Study of the Peripheral Nerve Fibers Myelin Structure Changes during Activation of Schwann Cell Acetylcholine Receptors. PLoS One. 2016;11(7):e0158083. doi: 10.1371/journal.pone.0158083 27455410 PMC4959681

[19] Gebrekidan MT, Knipfer C, Braeuer AS. Refinement of spectra using a deep neural network: Fully automated removal of noise and background. J Raman Spectrosc. 2021;52(3):723–36.

[20] Gebrekidan MT, Knipfer C, Stelzle F, Popp J, Will S, Braeuer A. A Shifted‐Excitation Raman Difference Spectroscopy (SERDS) evaluation strategy for the efficient isolation of Raman spectra from extreme fluorescence interference. J Raman Spectrosc. 2016;47(2):198–209.

[21] Matthies L, Gebrekidan MT, Tegtmeyer JF, Oetter N, Rohde M, Vollkommer T, et al. Optical diagnosis of oral cavity lesions by label-free Raman spectroscopy. Biomed Opt Express. 2021;12(2):836–51. doi: 10.1364/BOE.409456 33680545 PMC7901324

[22] Maher JR, Chuchuen O, Henderson MH, Kim S, Rinehart MT, Kashuba AD, et al. Co-localized confocal Raman spectroscopy and optical coherence tomography (CRS-OCT) for depth-resolved analyte detection in tissue. Biomed Opt Express. 2015;6(6):2022–35. doi: 10.1364/BOE.6.002022 26114026 PMC4473741

[23] Kim H, Drake BL, Park H. Multiclass classifiers based on dimension reduction with generalized LDA. Pattern Recogn. 2007;40(11):2939–45.

[24] Khan A, Farooq H. Principal component analysis-linear discriminant analysis feature extractor for pattern recognition. arXiv preprint arXiv:12041177. 2012.

[25] Bergstra J, Komer B, Eliasmith C, Yamins D, Cox DD. Hyperopt: a Python library for model selection and hyperparameter optimization. Comput Sci Discov. 2015;8(1):014008.

[26] Jo VY, Fletcher CD. WHO classification of soft tissue tumours: an update based on the 2013 (4th) edition. Pathology. 2014;46(2):95–104. doi: 10.1097/PAT.0000000000000050 24378391

[27] Bonnier F, Byrne HJ. Understanding the molecular information contained in principal component analysis of vibrational spectra of biological systems. Analyst. 2012;137(2):322–32. doi: 10.1039/c1an15821j 22114757

[28] Sigurdsson S, Philipsen PA, Hansen LK, Larsen J, Gniadecka M, Wulf H-C. Detection of skin cancer by classification of Raman spectra. IEEE transactions on biomedical engineering. 2004;51(10):1784–93. doi: 10.1109/TBME.2004.831538 15490825

[29] Talari ACS, Movasaghi Z, Rehman S, Rehman IU. Raman Spectroscopy of Biological Tissues. Appl Spectrosc Rev. 2015;50(1):46–111.

[30] Zhou Y, Liu CH, Sun Y, Pu Y, Boydston-White S, Liu Y, et al. Human brain cancer studied by resonance Raman spectroscopy. J Biomed Opt. 2012;17(11):116021. doi: 10.1117/1.JBO.17.11.116021 23154776 PMC3499405

[31] Krafft C, Neudert L, Simat T, Salzer R. Near infrared Raman spectra of human brain lipids. Spectrochim Acta A Mol Biomol Spectrosc. 2005;61(7):1529–35. doi: 10.1016/j.saa.2004.11.017 15820887

[32] Cheng WT, Liu MT, Liu HN, Lin SY. Micro-Raman spectroscopy used to identify and grade human skin pilomatrixoma. Microsc Res Tech. 2005;68(2):75–9. doi: 10.1002/jemt.20229 16228983

[33] Rodriguez FJ, Folpe AL, Giannini C, Perry A. Pathology of peripheral nerve sheath tumors: diagnostic overview and update on selected diagnostic problems. Acta Neuropathol. 2012;123(3):295–319. doi: 10.1007/s00401-012-0954-z 22327363 PMC3629555

[34] Ristow I, Madesta F, Well L, Shenas F, Wright F, Molwitz I, et al. Evaluation of magnetic resonance imaging-based radiomics characteristics for differentiation of benign and malignant peripheral nerve sheath tumors in neurofibromatosis type 1. Neuro Oncol. 2022;24(10):1790–8. doi: 10.1093/neuonc/noac100 35426432 PMC9527508

[35] Butler HJ, Ashton L, Bird B, Cinque G, Curtis K, Dorney J, et al. Using Raman spectroscopy to characterize biological materials. Nat Protoc. 2016;11(4):664–87. doi: 10.1038/nprot.2016.036 26963630

[36] Lyng FM, Traynor D, Nguyen TNQ, Meade AD, Rakib F, Al-Saady R, et al. Discrimination of breast cancer from benign tumours using Raman spectroscopy. PLoS One. 2019;14(2):e0212376. doi: 10.1371/journal.pone.0212376 30763392 PMC6375635

[37] Chen Y, Dai J, Zhou X, Liu Y, Zhang W, Peng G. Raman Spectroscopy Analysis of the Biochemical Characteristics of Molecules Associated with the Malignant Transformation of Gastric Mucosa. PLoS One. 2014;9(4):e93906. doi: 10.1371/journal.pone.0093906 24710050 PMC3977959

[38] Hollon T, Orringer DA. Label-free brain tumor imaging using Raman-based methods. J Neurooncol. 2021;151(3):393–402. doi: 10.1007/s11060-019-03380-z 33611706 PMC9333091

[39] Senft C, Bink A, Franz K, Vatter H, Gasser T, Seifert V. Intraoperative MRI guidance and extent of resection in glioma surgery: a randomised, controlled trial. Lancet Oncol. 2011;12(11):997–1003. doi: 10.1016/S1470-2045(11)70196-6 21868284

[40] Stummer W, Pichlmeier U, Meinel T, Wiestler OD, Zanella F, Reulen HJ. Fluorescence-guided surgery with 5-aminolevulinic acid for resection of malignant glioma: a randomised controlled multicentre phase III trial. Lancet Oncol. 2006;7(5):392–401. doi: 10.1016/S1470-2045(06)70665-9 16648043

[41] Poulon F, Mehidine H, Juchaux M, Varlet P, Devaux B, Pallud J, et al. Optical properties, spectral, and lifetime measurements of central nervous system tumors in humans. Sci Rep. 2017;7(1):13995. doi: 10.1038/s41598-017-14381-1 29070870 PMC5656602

[42] Whitney MA, Crisp JL, Nguyen LT, Friedman B, Gross LA, Steinbach P, et al. Fluorescent peptides highlight peripheral nerves during surgery in mice. Nat Biotechnol. 2011;29(4):352–6. doi: 10.1038/nbt.1764 21297616 PMC3364105

[43] Minamikawa T, Harada Y, Koizumi N, Okihara K, Kamoi K, Yanagisawa A, et al. Label-free detection of peripheral nerve tissues against adjacent tissues by spontaneous Raman microspectroscopy. Histochem Cell Biol. 2013;139(1):181–93. doi: 10.1007/s00418-012-1015-3 22892663

[44] Gao L, Zhou H, Thrall MJ, Li F, Yang Y, Wang Z, et al. Label-free high-resolution imaging of prostate glands and cavernous nerves using coherent anti-Stokes Raman scattering microscopy. Biomed Opt Express. 2011;2(4):915–26. doi: 10.1364/BOE.2.000915 21483613 PMC3072130

[45] Yadav R, Mukherjee S, Hermen M, Tan G, Maxfield FR, Webb WW, et al. Multiphoton microscopy of prostate and periprostatic neural tissue: a promising imaging technique for improving nerve-sparing prostatectomy. J Endourol. 2009;23(5):861–7. doi: 10.1089/end.2009.0221 19425823 PMC2841021

[46] Durand M, Jain M, Aggarwal A, Robinson BD, Srivastava A, Smith R, et al. Real-time in vivo periprostatic nerve tracking using multiphoton microscopy in a rat survival surgery model: a promising pre-clinical study for enhanced nerve-sparing surgery. BJU Int. 2015;116(3):478–86. doi: 10.1111/bju.12903 25124551

[47] Morisaki S, Ota C, Matsuda K, Kaku N, Fujiwara H, Oda R, et al. Application of Raman spectroscopy for visualizing biochemical changes during peripheral nerve injury in vitro and in vivo. J Biomed Opt. 2013;18(11):116011. doi: 10.1117/1.JBO.18.11.116011 24281358

[48] Kumamoto Y, Harada Y, Tanaka H, Takamatsu T. Rapid and accurate peripheral nerve imaging by multipoint Raman spectroscopy. Sci Rep. 2017;7(1):845. doi: 10.1038/s41598-017-00995-y 28405007 PMC5429797

[49] Rabah R, Weber R, Serhatkulu GK, Cao A, Dai H, Pandya A, et al. Diagnosis of neuroblastoma and ganglioneuroma using Raman spectroscopy. J Pediatr Surg. 2008;43(1):171–6. doi: 10.1016/j.jpedsurg.2007.09.040 18206477

[50] Wills H, Kast R, Stewart C, Rabah R, Pandya A, Poulik J, et al. Raman spectroscopy detects and distinguishes neuroblastoma and related tissues in fresh and (banked) frozen specimens. J Pediatr Surg. 2009;44(2):386–91. doi: 10.1016/j.jpedsurg.2008.10.095 19231540

[51] Lunter D, Klang V, Kocsis D, Varga-Medveczky Z, Berko S, Erdo F. Novel aspects of Raman spectroscopy in skin research. Exp Dermatol. 2022;31(9):1311–29. doi: 10.1111/exd.14645 35837832 PMC9545633

[52] Bratchenko IA, Bratchenko LA, Moryatov AA, Khristoforova YA, Artemyev DN, Myakinin OO, et al. In vivo diagnosis of skin cancer with a portable Raman spectroscopic device. Exp Dermatol. 2021;30(5):652–63. doi: 10.1111/exd.14301 33566431

[53] Shreve AP, Cherepy NJ, Mathies RA. Effective Rejection of Fluorescence Interference in Raman Spectroscopy Using a Shifted Excitation Difference Technique. Appl Spectrosc. 1992;46(4):707–11.

[54] Noack K, Eskofier B, Kiefer J, Dilk C, Bilow G, Schirmer M, et al. Combined shifted-excitation Raman difference spectroscopy and support vector regression for monitoring the algal production of complex polysaccharides. Analyst. 2013;138(19):5639–46. doi: 10.1039/c3an01158e 23905163

[55] Kiefer J. Instantaneous Shifted‐Excitation Raman Difference Spectroscopy (iSERDS). J Raman Spectrosc. 2014;45(10):980–3.

[56] Gebrekidan MT, Erber R, Hartmann A, Fasching PA, Emons J, Beckmann MW, et al. Breast tumor analysis using Shifted-Excitation Raman Difference Spectroscopy (SERDS). Technol Cancer Res Treat. 2018;17:1–11. doi: 10.1177/1533033818782532 29991340 PMC6048663

[57] Shaik TA, Baria E, Wang X, Korinth F, Lagarto JL, Höppener C, et al. Structural and Biochemical Changes in Pericardium upon Genipin Cross-Linking Investigated Using Nondestructive and Label-Free Imaging Techniques. Anal Chem. 2022;94(3):1575–84. doi: 10.1021/acs.analchem.1c03348 35015512

[58] Knipfer C, Motz J, Adler W, Brunner K, Gebrekidan MT, Hankel R, et al. Raman difference spectroscopy: a non-invasive method for identification of oral squamous cell carcinoma. Biomedical Optics Express. 2014;5(9):3252–65. doi: 10.1364/BOE.5.003252 25401036 PMC4230857

[59] Kasha M. Characterization of electronic transitions in complex molecules. Discuss Faraday Soc. 1950;9:14–9.

[60] da Silva Martins MA, Ribeiro DG, Pereira Dos Santos EA, Martin AA, Fontes A, da Silva Martinho H. Shifted-Excitation Raman Difference Spectroscopy for in vitro and in vivo biological samples analysis. Biomed Opt Express. 2010;1(2):617–26. doi: 10.1364/BOE.1.000617 21258495 PMC3018003

[61] Dochow S, Bergner N, Matthäus C, Praveen BB, Ashok PC, Mazilu M, et al. Etaloning, fluorescence and ambient light suppression by modulated wavelength Raman spectroscopy. Biomed Spectrosc Imaging. 2012;1(4):383–9.

[62] Lieber CA, Mahadevan-Jansen A. Automated method for subtraction of fluorescence from biological Raman spectra. Appl Spectrosc. 2003;57(11):1363–7. doi: 10.1366/000370203322554518 14658149

[63] Baek SJ, Park A, Ahn YJ, Choo J. Baseline correction using asymmetrically reweighted penalized least squares smoothing. Analyst. 2015;140(1):250–7. doi: 10.1039/c4an01061b 25382860

[64] Lipiainen T, Pessi J, Movahedi P, Koivistoinen J, Kurki L, Tenhunen M, et al. Time-gated Raman spectroscopy for quantitative determination of solid-state forms of fluorescent pharmaceuticals. Anal Chem. 2018;90(7):4832–9. doi: 10.1021/acs.analchem.8b00298 29513001 PMC6150637

[65] Korinth F, Mondol AS, Stiebing C, Schie IW, Krafft C, Popp J. New methodology to process shifted excitation Raman difference spectroscopy data: a case study of pollen classification. Sci Rep. 2020;10(1):11215. doi: 10.1038/s41598-020-67897-4 32641779 PMC7343813

[66] Sowoidnich K, Maiwald M, Sumpf B, Towrie M, Matousek P, editors. Charge-shifting optical lock-in detection with shifted excitation Raman difference spectroscopy for the analysis of fluorescent heterogeneous samples. ProcSPIE; 2020.

[67] Korinth F, Schmalzlin E, Stiebing C, Urrutia T, Micheva G, Sandin C, et al. Wide Field Spectral Imaging with Shifted Excitation Raman Difference Spectroscopy Using the Nod and Shuffle Technique. Sensors (Basel). 2020;20(23). doi: 10.3390/s20236723 33255459 PMC7727830

[68] Ronneberger O, Fischer P, Brox T, editors. U-net: Convolutional networks for biomedical image segmentation. Medical Image Computing and Computer-Assisted Intervention–MICCAI 2015: 18th International Conference, Munich, Germany, October 5–9, 2015, Proceedings, Part III 18; 2015: Springer.

[69] Hollon TC, Pandian B, Adapa AR, Urias E, Save AV, Khalsa SSS, et al. Near real-time intraoperative brain tumor diagnosis using stimulated Raman histology and deep neural networks. Nat Med. 2020;26(1):52–8. doi: 10.1038/s41591-019-0715-9 31907460 PMC6960329

[70] Huang Z, McWilliams A, Lam S, English J, McLean DI, Lui H, et al. Effect of formalin fixation on the near-infrared Raman spectroscopy of normal and cancerous human bronchial tissues. Int J Oncol. 2003;23(3):649–55. 12888900

[71] Fiedler IAK, Casanova M, Keplinger T, Busse B, Muller R. Effect of short-term formaldehyde fixation on Raman spectral parameters of bone quality. J Biomed Opt. 2018;23(11):1–6. doi: 10.1117/1.JBO.23.11.116504 30499261

[72] Maiwald M, Muller A, Sumpf B, Erbert G, Trankle G. Capability of shifted excitation Raman difference spectroscopy under ambient daylight. Appl Opt. 2015;54(17):5520–4. doi: 10.1364/AO.54.005520 26192855

[73] Vohra P, Strobbia P, Ngo HT, Lee WT, Vo-Dinh T. Rapid Nanophotonics Assay for head and neck cancer diagnosis. Sci Rep. 2018;8(1):11410. doi: 10.1038/s41598-018-29428-0 30061592 PMC6065408

[74] Dukes PV, Strobbia P, Ngo HT, Odion RA, Rocke D, Lee WT, et al. Plasmonic assay for amplification-free cancer biomarkers detection in clinical tissue samples. Anal Chim Acta. 2020;1139:111–8. doi: 10.1016/j.aca.2020.09.003 33190693

[75] Peltonen J, Penttinen R, Larjava H, Aho HJ. Collagens in neurofibromas and neurofibroma cell cultures. Ann N Y Acad Sci. 1986;486:260–70. doi: 10.1111/j.1749-6632.1986.tb48079.x 3105391

